# Intermittent Hypoxia Alters Gene Expression in Peripheral Blood Mononuclear Cells of Healthy Volunteers

**DOI:** 10.1371/journal.pone.0144725

**Published:** 2015-12-14

**Authors:** Vsevolod Y. Polotsky, Shannon Bevans-Fonti, Dmitry N. Grigoryev, Naresh M. Punjabi

**Affiliations:** 1 Department of Medicine, Division of Pulmonary and Critical Care Medicine, Johns Hopkins University School of Medicine, Baltimore, Maryland, United States of America; 2 Department of Medicine, Division of Allergy and Clinical Immunology, Johns Hopkins University School of Medicine, Baltimore, Maryland, United States of America; 3 Department of Epidemiology, Bloomberg School of Public Health, Johns Hopkins University, Baltimore, Maryland, United States of America; Virginia Polytechnic Institute and State University, UNITED STATES

## Abstract

Obstructive sleep apnea is associated with high cardiovascular morbidity and mortality. Intermittent hypoxia of obstructive sleep apnea is implicated in the development and progression of insulin resistance and atherosclerosis, which have been attributed to systemic inflammation. Intermittent hypoxia leads to pro-inflammatory gene up-regulation in cell culture, but the effects of intermittent hypoxia on gene expression in humans have not been elucidated. A cross-over study was performed exposing eight healthy men to intermittent hypoxia or control conditions for five hours with peripheral blood mononuclear cell isolation before and after exposures. Total RNA was isolated followed by gene microarrays and confirmatory real time reverse transcriptase PCR. Intermittent hypoxia led to greater than two fold up-regulation of the pro-inflammatory gene toll receptor 2 (TLR2), which was not increased in the control exposure. We hypothesize that up-regulation of TLR2 by intermittent hypoxia may lead to systemic inflammation, insulin resistance and atherosclerosis in patients with obstructive sleep apnea.

## Introduction

Obstructive Sleep Apnea (OSA) is characterized by recurrent collapse of the upper airway during sleep leading to intermittent hypoxemia and recurrent arousals from sleep. [1] It is now well established that OSA is associated with incident hypertension, cardiovascular disease, and all-cause mortality.[2–4] Although underlying mechanisms that explicate the increase in cardiovascular risk are not well defined, a commonly accepted hypotheses is that recurrent exposure to intermittent hypoxemia induces low grade systemic inflammation [5–9] that, in turn, can promote atherosclerosis and the development of insulin resistance. However, the putative links between intermittent hypoxemia and systemic inflammation are not well defined.

Over the last decade, gene microarray analyses have provided novel insights into intermediate mechanisms for a number of chronic conditions. In fact, utilizing whole blood or peripheral blood mononuclear cells (PBMC) RNA, gene microarrays have demonstrated up-regulation of pathways of oxidative stress and inflammation in adult and pediatric OSA samples.[10–12] While pioneering, the available studies to date are limited by inevitable substantial variability in the phenotype across patients with OSA. Genomic studies that have used cell culture and animal models have shown that exposure to intermittent hypoxia up-regulates signaling pathways for cardiac hypertrophy in the myocardium, [13] induces genes responsible for lipid biosynthesis in the liver [14] and activates pro-inflammatory genes in immortal cell lines [5] and in the endothelium.[8,15] In contrast, the acute effects of intermittent hypoxia on gene expression in humans have not been previously examined. The current study sought to evaluate PBMC gene expression profiles induced by intermittent hypoxia in healthy volunteers. To identify changes specifically induced by intermittent hypoxia, gene expression was examined in a cohort of normal subjects before and after exposure to intermittent hypoxia or air (control conditions). Moreover, to distinguish the effects of intermittent hypoxia from sleep fragmentation, the focus of the current study was to assess the effects during wakefulness and not sleep. It was hypothesized that exposure to intermittent hypoxia for as short as 5-hours will induce pro-inflammatory genes.

## Methods

### Human subjects

Healthy adult men (n = 8) were recruited from local community as previously described.[16] Exclusion criteria included a history of any respiratory illness, hypertension, hepatic, renal, cardiovascular, neurologic or a hematologic disorder, habitual sleep duration < 7 h, any circadian sleep disorder and current smoking.[16] In addition, a full montage polysomnogram excluded volunteers with undiagnosed obstructive sleep apnea. A mean age was 24.3 years (range 18–35 yr) and the body mass index was 25.8 ± 1.7 kg/m^2^, which is considered overweight.[17] Since 68.8% of the adult U.S. population is overweight or obese, [18] our volunteers adequately represented an average healthy U.S. adult. The study has been conducted according to the principles expressed in the Declaration of Helsinki. Written informed consents have been obtained from the participants. The consent forms signed by the participants have been scanned and stored on the secure drive. The study and the consent procedure have been approved by the Johns Hopkins University Institutional Review Board (IRB) 5.

### Study protocol

**Fig 1:**
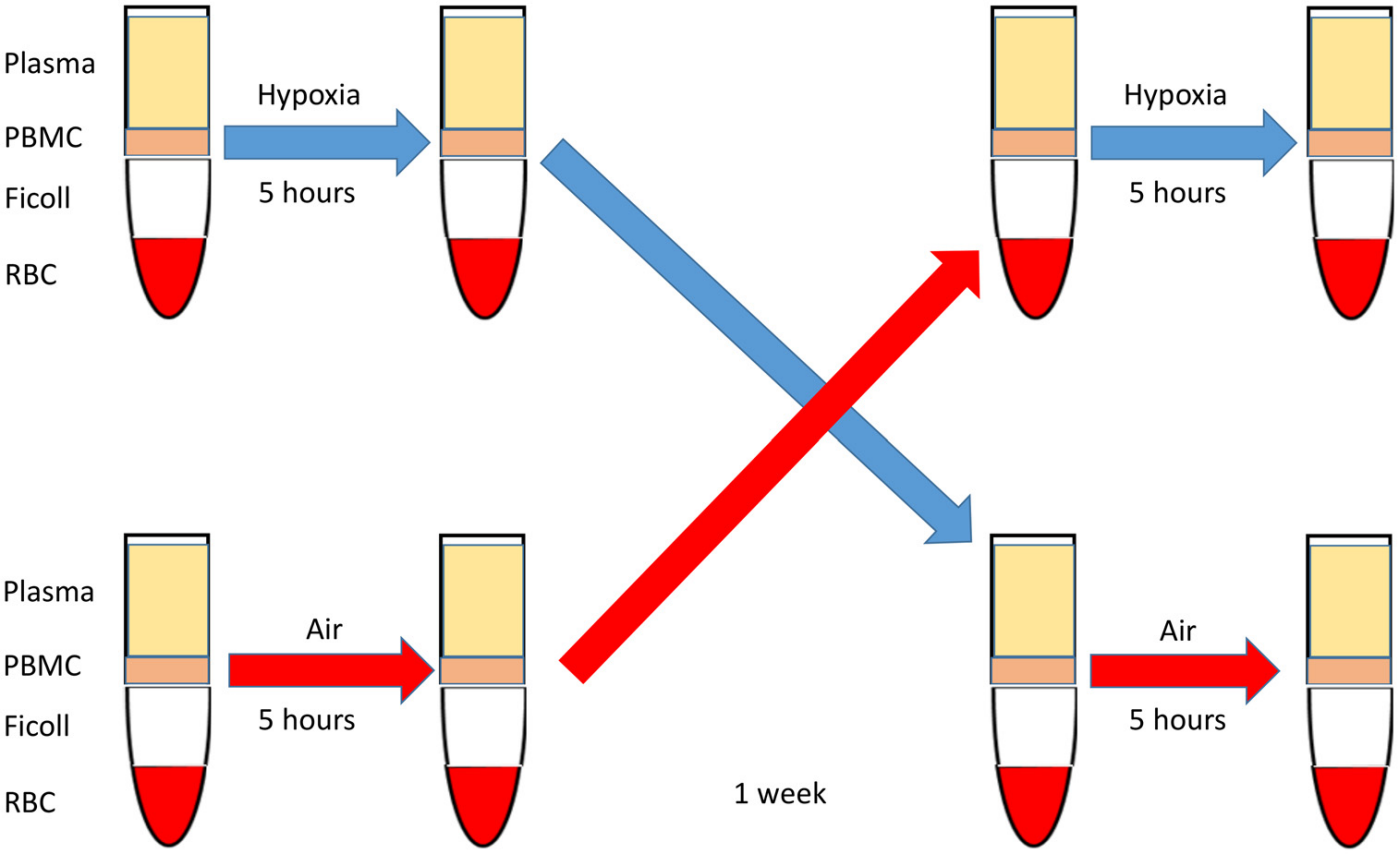
Cross-over study design. Healthy volunteers underwent phlebotomy in the antecubital fossa and whole blood (15 ml) was collected in the ethylenediaminetetraacetic acid (EDTA) coated tube. The volunteers were exposed to intermittent hypoxia or control air conditions (see Methods) for 5 hours followed by whole blood collection. In one week, the volunteers underwent phlebotomy again and had an alternative exposure followed by whole blood collection. Peripheral blood mononuclear cells (PBMC, lymphocytes and monocytes) were isolated immediately after blood collection using Ficoll-Hypaque solution. RBC, red blood cells.

A cross-over study was conducted in healthy volunteers (n = 8) who were exposed to intermittent hypoxia or control conditions in a randomized fashion for 5 hours while awake as previously described (Fig 1).[16] Briefly, the study was performed in a single-blind fashion and consisted of two days with an interval of one week. On one of the days, the subject was exposed to intermittent hypoxia for 5 h, from 8 am to 1 pm, and on the other day the subject was exposed to ambient air for 5 h, from 8 am to 1 pm, using a similar set up as a control. A full face mask was applied, and the inspiratory valve was attached to a three-way Hans-Rudolph valve so that inspiration could be from one of two pressurized cylinders with ambient air (21% O_2_) or hypoxic gas (95% N_2_ and 5% O_2_). Intermittent hypoxia was induced by inspiration of the hypoxic (95% N_2_ and 5% O_2_) gas until the oxyhemoglobin saturation dropped to 85% followed by reoxygenation to the baseline, resulting in ~25 hypoxic events/h. In control condition, the hypoxic gas was substituted with air. Phlebotomy was performed in the antecubital fossa and whole blood (15 ml) was collected immediately prior and after the exposure.

### PBMC isolation

Whole blood (15 ml) was anticoagulated with ethylenediaminetetraacetic acid (EDTA), mixed with phosphate-buffered saline (PBS) 1:1, layered over Ficoll-Hypaque solution (Pharmacia, Biotech AB, Uppsala, Sweden) (20 ml) and centrifuged at 400 x g for 40 min according to the manufacturer’s instructions. PBMCs were isolated, washed in ice-cold PBS and snap-frozen at -80°C.

### Gene microarrays

Microarray studies were performed in four subjects before and after intermittent hypoxia or control exposures. Total RNA was isolated from PBMCs using the Trizol Reagent method (Invitrogen, Carlsbad, California 92008, cat. no. 15596–026) with subsequent RNEasy clean up (Qiagen, Valencia, CA 913555, cat. no. 74104). 0.5 μg of total RNA from each sample were labelled using the Illumina TotalPrep RNA Amplification Kit (Ambion, Austin, TX 78744–1832, cat. no. IL1791). RNA was converted into double-stranded cDNA using an oligo-dT primer containing the T7 RNA polymerase promoter. Single stranded RNA (cRNA) was from double-stranded cDNA in an *in vitro* transcription reaction. cRNA was labelled by incorporating biotin-16-UTP. 0.85 ug of biotin-labelled cRNA was hybridized (16 hours) to Illumina’s Sentrix HumanRef-8 Expression BeadChips (Illumina, San Diego, CA 92121–1975, cat.no. 11201828). The hybridized biotinylated cRNA was detected with streptavidin-Cy3 and quantified with Illumina’s BeadStation 500GX Genetic Analysis Systems scanners. Preliminary analysis of the scanned data was performed using Illumina BeadStudio software. Resulting digitized matrix was processed by modified for Illumina platform approach described previously.[19] The significant hybridization signals were defined based on the BeadStudio identification ratio IR>0.95. The chip background and brightness were computed using high quartile and whole set of hybridization signals that fell below IR<0.95. The expression data was stratified by experimental conditions and hybridization of each transcript was evaluated. The transcripts that were detectable by BeadStudio (IR>0.95) and produced signal at least twice as high as that of background in at least 3 out of 4 hybridizations in any given experimental condition were considered truly expressed. The signal intensity values of these transcripts from each chip were increased by corresponding to a given chip background value (background adjustment) and divided by a chip brightness coefficient (normalization).

### Real time PCR

Real-time reverse-transcriptase PCR (RT-PCR) was performed in all eight subjects before and after intermittent hypoxia and control exposures. Total RNA was extracted using Trizol and cDNA was synthesized using Advantage RT for PCR kit from Clontech (Palo Alto, CA). Real-time reverse-transcriptase PCR (RT-PCR) was performed with primers from Invitrogen (Carlsbad, CA). The sequences of primers and probes for 18s were previously described.[20] The sequences of primers were designed based on the GeneBank sequences: for toll receptor 2 (TLR-2)[NM_003264.3], forward 5’-CGGAAGTGCTGTCCTGTGACATTC[FAM]G-3’, reverse 5’- GGCCAGCAAATTACCTGTGTGA-3’; for chemokine (C-C motif) receptor 2 (CCR2) [NM_000647], forward 5’- CGGCCTGAGTAACTGTGA AAGC -3’, reverse 5’- CGCAAAGAGTCTCTGTCACCTG[FAM]G-3’; for hemoglobin alpha 1 (HBA1) [NM_000558], forward 5’- CGGGCCACCAAGACCTACTTCC [FAM]G-3’, reverse 5’-CTTGCCGTGGCCCTTAACCT-3’. The mRNA expression levels were normalized to 18s rRNA concentrations using the following formula: Gene of interest/18S = 2 ^Ct(18S)-Ct(Gene of Interest)^.

### ELISA

The enzyme-linked immunosorbent assay (ELISA) was performed in EDTA plasma samples of all eight subjects before and after intermittent hypoxia and control exposures using a human interleukin 8 (IL-8) kit from R&D Systems (Minneapolis, MN).

### Statistical analysis

Significance Analysis of Microarrays (SAM 2.20) was conducted using 1000 permutation of 4 control and 4 treated PBMC samples without application of arbitrary restrictions. Genes with 2.0 fold change and 5% false discovery rate (q) were considered significantly affected by intermittent hypoxia. All values obtained in PCR are reported as means ± SEM. Comparisons between baseline and 5 hrs data points after IH and control exposures were performed using repeated-measures ANOVA. A p-value ≤ 0.05 was considered significant.

## Results

In four healthy human volunteers, exposure to intermittent hypoxia up-regulated 20 genes and down-regulated 7 genes out of 12291 genes, which were examined with the Illumina’s Sentrix HumanRef-8 Expression BeadChip (Table 1). In contrast, exposure of the same subjects to control conditions did not lead to marked induction of any genes (Table 2), and 29 genes were down-regulated. Among genes induced by intermittent hypoxia, we detected increases in pro-atherogenic chemokine receptor CCR-2 [21] and TLR-2 and a number of apoptotic factors such as PDCD4 and APAF1 (Table 1). Among genes down-regulated by intermittent hypoxia were hemoglobin transcripts, including hemoglobin delta, alpha 1 and 2, HBD, HBA1 and HBA2, respectively (Table 1). However, the hemoglobin transcripts were similarly down-regulated after exposure to control condition suggesting that the changes in gene expression were not specific to the hypoxic stimulus (Table 2). Exposure to control conditions decreased expression of IL-8 and IL-8 receptor in PBMCs (Table 2), which did not occur during intermittent hypoxia (Table 1). However, plasma levels of IL-8 were undetectable, regardless of conditions.

**Table 1:** Genes Differentially Regulated in Peripheral Blood Mononuclear Cells after Exposure to Intermittent Hypoxia for 5 hrs Compared to Baseline.

N	Target ID	Gene Name	Fold Change	q-value (%)
1	GI_21361115-S	chondroitin sulfate proteoglycan 2 (versican) (CSPG2)	**3.26**	0.216
2	GI_13325059-S	cytochrome P450, family 1, subfamily B, polypeptide 1 (CYP1B1)	**2.74**	0.216
3	GI_15451896-I	chemokine (C-C motif) receptor 2 (CCR2), transcript variant A	**2.55**	0.216
4	GI_31377778-S	serine/threonine kinase 38 (STK38)	**2.47**	0.216
5	GI_34304340-A	programmed cell death 4 (PDCD4), transcript variant 1	**2.44**	0.216
6	GI_42659661-S	macrophage expressed gene 1 (MPEG1)	**2.44**	0.216
7	GI_29171722-A	G protein-coupled receptor 86 (GPR86), transcript variant 2	**2.38**	0.216
8	GI_28416432-S	immunity associated protein 4 (HIMAP4)	**2.32**	0.216
9	GI_32483362-A	apoptotic protease activating factor (APAF1), transcript variant 5	**2.31**	0.216
10	GI_32261305-S	IQ motif containing GTPase activating protein 1 (IQGAP1)	**2.24**	0.216
11	GI_5730074-S	fibrinogen-like 2 (FGL2)	**2.16**	0.216
12	GI_32307149-A	O-linked N-acetylglucosamine (GlcNAc) transferase (OGT), transcript variant 2	**2.15**	0.216
13	GI_21536438-A	F-box and leucine-rich repeat protein 5 (FBXL5), transcript variant 1	**2.13**	0.216
14	GI_22027524-S	Rac/Cdc42 guanine nucleotide exchange factor (GEF) 6 (ARHGEF6)	**2.12**	0.216
15	GI_4506516-S	regulator of G-protein signalling 2, 24kDa (RGS2)	**2.11**	0.216
16	GI_19718733-S	toll-like receptor 2 (TLR2)	**2.06**	0.216
17	GI_35493781-A	ring finger protein 19 (RNF19), transcript variant 2	**2.05**	0.216
18	GI_33667050-S	polyposis locus protein 1 (DP1)	**2.04**	0.216
19	GI_33589822-S	pyruvate dehydrogenase kinase, isoenzyme 4 (PDK4)	**2.01**	0.216
20	GI_32698701-S	HECT domain containing 1 (HECTD1)	**2.01**	0.216
21	GI_21071025-S	histone 1, H1c (HIST1H1C)	**-2.05**	2.647
22	GI_28302128-S	hemoglobin, beta (HBB)	**-2.16**	0.216
23	GI_4557676-S	integrin, beta 3 (platelet glycoprotein IIIa, antigen CD61) (ITGB3)	**-2.17**	0.730
24	GI_38455401-S	lipocalin 2 (oncogene 24p3) (LCN2)	**-2.50**	0.730
25	GI_6633803-S	hemoglobin, delta (HBD)	**-2.57**	0.216
26	GI_14043068-S	hemoglobin, alpha 2 (HBA2)	**-3.07**	0.216
27	GI_14456711-S	hemoglobin, alpha 1 (HBA1)	**-3.23**	0.216

**Table 2:** Genes Differentially Regulated in Peripheral Blood Mononuclear Cells after Exposure to Control Conditions for 5 hours Compared to Baseline

N	Target ID	Gene Name	Fold Change	q-value (%)
1	GI_42658769-S	hypothetical gene supported by BC062364; BX647289 (LOC401457)	**-2.01**	0.356
2	GI_31982886-S	GATA binding protein 2 (GATA2)	**-2.04**	0.356
3	GI_28610153-S	interleukin 8 (IL8)	**-2.05**	0.356
4	GI_4504152-S	chemokine (C-X-C motif) ligand 1 (CXCL1)	**-2.08**	0.356
5	GI_5174662-S	S100 calcium binding protein P (S100P)	**-2.10**	0.356
6	GI_7706179-S	erythroid associated factor (ERAF)	**-2.16**	0.356
7	GI_38455401-S	lipocalin 2 (oncogene 24p3) (LCN2)	**-2.17**	0.356
8	GI_13787192-S	alkaline phosphatase, liver/bone/kidney (ALPL)	**-2.19**	0.356
9	GI_7706149-A	mitochondrial solute carrier protein (MSCP)	**-2.24**	0.356
10	GI_23110992-S	membrane-spanning 4-domains, subfamily A, member 3 (MS4A3)	**-2.24**	0.356
11	GI_31657130-S	protease inhibitor 3, skin-derived (SKALP) (PI3)	**-2.25**	0.356
12	GI_30581169-A	chemokine (C-C motif) receptor 3 (CCR3), transcript variant 2	**-2.27**	0.356
13	GI_29171680-S	interleukin 8 receptor, beta (IL8RB)	**-2.28**	0.356
14	GI_31563435-S	chemokine-like factor super family 2 (CKLFSF2)	**-2.28**	0.356
15	GI_4826835-S	matrix metalloproteinase 9 (MMP9)	**-2.30**	0.356
16	GI_39753969-S	cathelicidin antimicrobial peptide (CAMP)	**-2.37**	0.356
17	GI_28302128-S	hemoglobin, beta (HBB)	**-2.37**	0.356
18	GI_12621916-S	defensin, alpha 3, neutrophil-specific (DEFA3)	**-2.42**	0.356
19	GI_21071007-S	transcobalamin I (TCN1)	**-2.50**	0.356
20	GI_4505042-S	lactotransferrin (LTF)	**-2.54**	0.356
21	GI_4557298-S	aminolevulinate, delta-, synthase 2 (ALAS2)	**-2.57**	0.356
22	GI_28302130-S	hemoglobin, gamma A (HBG1)	**-2.64**	0.356
23	GI_29171679-S	interleukin 8 receptor, alpha (IL8RA)	**-2.65**	0.356
24	GI_28302132-S	hemoglobin, gamma G (HBG2)	**-2.66**	0.356
25	GI_13027808-A	matrix metalloproteinase 25 (MMP25), transcript variant 2	**-2.67**	0.356
26	GI_21536462-I	matrix metalloproteinase 25 (MMP25), transcript variant 1	**-3.08**	0.356
27	GI_6633803-S	hemoglobin, delta (HBD)	**-3.21**	0.356
28	GI_14043068-S	hemoglobin, alpha 2 (HBA2)	**-3.83**	0.356
29	GI_14456711-S	hemoglobin, alpha 1 (HBA1)	**-4.18**	0.356

The data discussed in this publication have been deposited in NCBI’s Gene Expression Omnibus [22,23] and are accessible through GEO series accession no. GSE71356 http://www.ncbi.nlm.nih.gov/geo/query/acc.cgi?acc=GSE71356.

**Fig 2:**
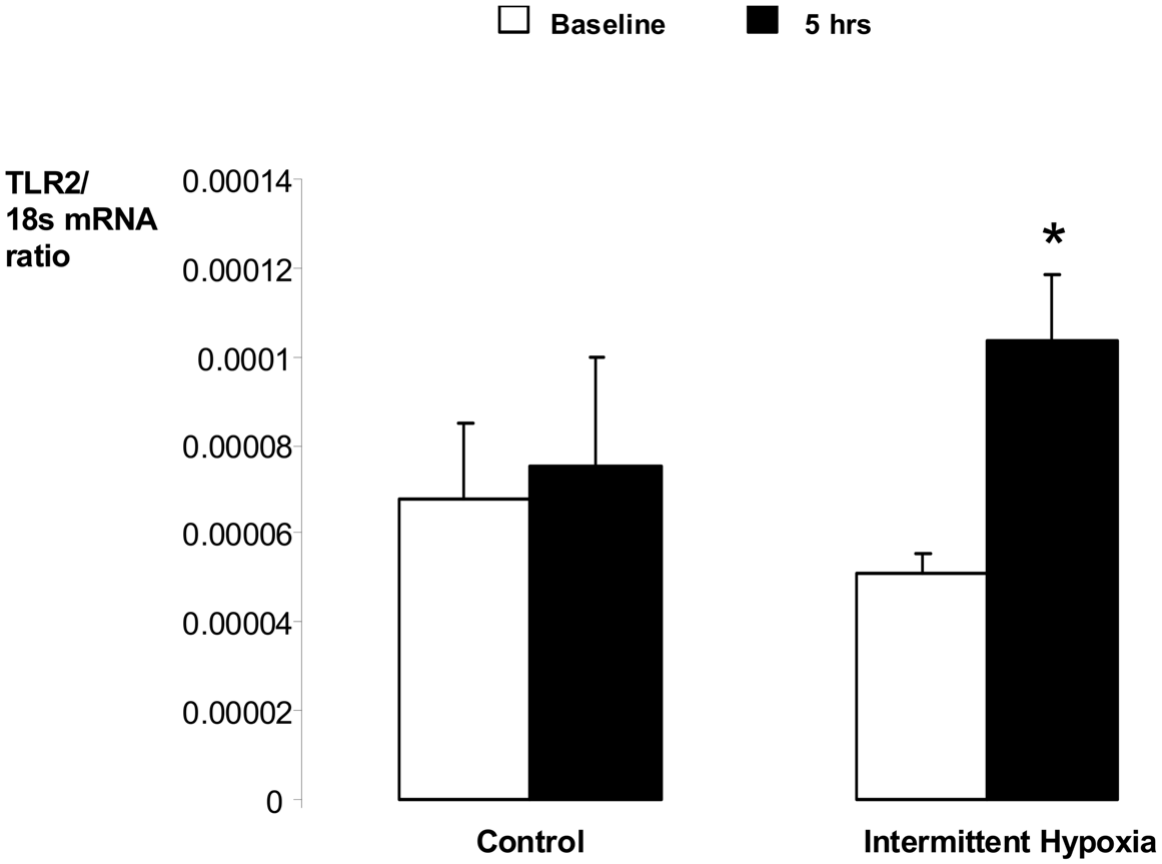
Intermittent hypoxia increased expression of toll-like receptor 2 in peripheral blood mononuclear cells. Expression of toll-like receptor two (TLR2) in peripheral blood mononuclear cells of healthy volunteers was measured during daytime exposure to intermittent hypoxia or control conditions for 5 hours and compared to baseline by real time PCR. The results are expressed as ratios to 18s. * denotes p < 0.05 for the difference between baseline and 5 hours data points.

Expression of selected genes has been examined in real-time PCR in eight healthy volunteers, including four volunteers, whose PBMC were studied by gene arrays. According to the PCR data, there were no differences in the levels of expression of CCR2 and HBA1 between baseline and IH (not shown). In contrast, 5 hours of intermittent hypoxia resulted in a 2.0 ± 0.3-fold increase in TLR2 mRNA levels, which did not occur at control conditions (Fig 2).

## Discussion

In the current study, healthy awake volunteers were exposed to acute intermittent hypoxia for 5 hours to mimic the oxygen profile of patients with moderate to severe OSA or to comparable control conditions. Gene expression profiles were examine in PBMCs before and after the exposure. The primary finding is that intermittent hypoxia caused a 2 fold induction of TLR2, an important gene of systemic and vascular inflammation.

Toll receptors are the family of pattern recognition receptors, which were initially described as the first line of defense against pathogens.[24] TLRs bind to different component of microorganisms including lipopeptide, double-stranded RNA, lipopolysaccharides, flagellin, etc. TLRs act via myeloid differentiation primary-response gene 88 (MyD88) to recruit downstream kinases and activate nuclear factor kappa B (NF-kB). TLR-driven induction of NF-kB results in production of pro-inflammatory cytokines, including TNFα, interleukin 1, interleukin 6 (IL-6), and IL-8, which play a role in the development of atherosclerosis and insulin resistance.[25–27] TLR2 deficiency inhibits the progression of atherosclerosis in *ApoE*-deficient mice and improves structural stability of the plaque, whereas TLR2 ligand specific activation accelerates atherosclerosis.[28] TLR2 agonists induce insulin resistance in 3T3-L1 adipocytes.[29] Furthermore, inhibition of TLR2 expression with antisense oligonucleotides improved insulin sensitivity in muscle and white adipose tissue in mice with diet-induced obesity.[30] Thus, up-regulation of TLR2 may lead to systemic inflammation, insulin resistance and atherosclerosis.

OSA has been associated with systemic inflammation with increased circulating levels of pro-inflammatory cytokines, including TNF-α, IL-6 and IL-8, independent of obesity.[5–7,31] Our data showing decreases in IL-8 and its receptor gene expression during control exposure (Table 2) are consistent with circadian variations of IL-8 levels peaking in the early morning hours.[32] This circadian decline was no longer present during intermittent hypoxia suggesting that IL-8 may have been up-regulated. Of note, short-term exposure of human endothelial cells to intermittent hypoxia is known to up-regulate IL-8 gene expression.[8] Studies in animal models of intermittent hypoxia showed that chronic intermittent hypoxia up-regulates NF-kB and downstream pro-inflammatory cytokines in multiple tissues.[33–36] Moreover, it has also been demonstrated that chronic intermittent hypoxia causes atherosclerosis in C57BL/6J mice that are fed a high fat diet.[20;36] In vitro studies have demonstrated that hypoxia can lead to a robust induction of TLR2 in various cell types acting via a master regulator of hypoxic responses, hypoxia inducible factor 1 (HIF-1).[37] It is conceivable that induction of TLR-2 by intermittent nocturnal hypoxemia contributes to the progression of systemic inflammation and atherosclerosis in patients with OSA. Indeed, nocturnal oxyhemoglobin desaturation in patients with OSA has been independently associated with atherosclerosis as assessed by increased carotid artery intima-media thickness [38;39] that can be reversed with continuous positive airway pressure therapy.[40] Given that exposure to intermittent hypoxia in healthy subjects, as done in the current study, can induce insulin resistance, [16] it is certainly possible that systemic induction of TLR2 may also lead to the development of insulin resistance in OSA.

The current study has several caveats. First, TLR2 protein levels were not measured and which cell type increases TLR2 expression was not assessed. We were unable to collect sufficient amount of blood from volunteers to perform these additional measurements due to ethical limitations of the human study. Nevertheless, transcriptional regulation is an important element of TLR functioning and up-regulation of TLR2 may lead to inflammatory response regardless whether it occurs in lymphocytes or monocytes.[41;42] Second, expression of pro-inflammatory cytokines downstream of TLR2 was not characterized with exception of IL-8, plasma levels of which remained undetectable throughout hypoxic and control exposures. Plasma IL-8 levels in healthy young adults is frequently below the sensitivity threshold of the ELISA assay.[43;44] The 5 hour exposure to intermittent hypoxia may not be long enough to induce measurable changes in cytokine protein levels.

In conclusion, we have demonstrated that TLR2 was one of 20 genes induced by exposure to intermittent hypoxia in healthy volunteers and were able to validate our data by real time PCR in a larger cohort. Thus, the current study suggests that hypoxic induction of TLR2 could be one of the mechanisms leading to systemic inflammation, atherosclerosis and insulin resistance in OSA.
